# Characterisation of a cysteine protease from poultry red mites and its potential use as a vaccine for chickens

**DOI:** 10.1051/parasite/2021005

**Published:** 2021-02-03

**Authors:** Shiro Murata, Ayaka Taniguchi, Masayoshi Isezaki, Sotaro Fujisawa, Eishi Sakai, Akira Taneno, Osamu Ichii, Takuya Ito, Naoya Maekawa, Tomohiro Okagawa, Satoru Konnai, Kazuhiko Ohashi

**Affiliations:** 1 Department of Disease Control, Faculty of Veterinary Medicine, Hokkaido University Kita-18, Nishi-9 Kita-ku Sapporo 060-0818 Japan; 2 Department of Advanced Pharmaceutics, Faculty of Veterinary Medicine, Hokkaido University Kita-18, Nishi-9 Kita-ku Sapporo 060-0818 Japan; 3 Vaxxinova Japan K.K. 1-24-8 Hamamatsucho Minato-ku Tokyo 105-0013 Japan; 4 Department of Basic Veterinary Sciences, Faculty of Veterinary Medicine, Hokkaido University Kita-18, Nishi-9 Kita-ku Sapporo 060-0818 Japan; 5 Hokkaido Institute of Public Health Kita-19, Nishi-12 Kita-ku Sapporo 060-0819 Japan

**Keywords:** Poultry red mite, Cysteine protease, Cathepsin L, Vaccine candidate, Deg-CPR-1, *Dermanyssus gallinae*

## Abstract

Poultry red mites (PRMs, *Dermanyssus gallinae*) are ectoparasites that negatively affect farmed chickens, leading to serious economic losses worldwide. Acaricides have been used to control PRMs in poultry houses. However, some PRMs have developed resistance to acaricides, and therefore different approaches are required to manage the problems caused by PRMs. Vaccination of chickens is one of the methods being considered to reduce the number of PRMs in poultry houses. In a previous study, a cysteine protease, Deg-CPR-1, was identified as a candidate vaccine against PRMs distributed in Europe. In this study, we investigated the characteristics of Deg-CPR-1. A phylogenetic analysis revealed that Deg-CPR-1 is closely related to the digestive cysteine proteases of other mite species, and it was classified into a cluster different from that of chicken cathepsins. Deg-CPR-1 of PRMs in Japan has an amino acid substitution compared with that of PRMs in Europe, but it showed efficacy as a vaccine, consistent with previous findings. Deg-CPR-1 exhibited cathepsin L-like enzyme activity. In addition, the *Deg-CPR-1* mRNA was expressed in the midgut and in all stages of PRMs that feed on blood. These results imply that Deg-CPR-1 in the midgut may have important functions in physiological processes, and the inhibition of its expression may contribute to the efficacy of a Deg-CPR-1-based vaccine. Further research is required to fully understand the mechanisms of vaccine efficacy.

## Abstract (fr)

Les acariens communément appelés poux rouges de la volaille (PRV, *Dermanyssus gallinae*) sont des ectoparasites qui affectent négativement les poulets d’élevage, entraînant de graves pertes économiques au niveau mondial. Des acaricides ont été utilisés pour contrôler les PRV dans les poulaillers. Cependant, certains PRV ont développé une résistance aux acaricides, et par conséquent, différentes approches sont nécessaires pour gérer les problèmes qu’ils causent. La vaccination des poulets est l’une des méthodes envisagées pour réduire le nombre de PRV dans les poulaillers. Dans une étude précédente, une cystéine protéase, Deg-CPR-1, a été identifiée comme un vaccin candidat contre les PRV distribués en Europe. Dans cette étude, nous avons étudié les caractéristiques de Deg-CPR-1. L’analyse phylogénétique a révélé que Deg-CPR-1 est étroitement liée aux cystéine protéases digestives d’autres espèces d’acariens, et elle a été classée dans un groupe différent de celui des cathepsines de poulet. La Deg-CPR-1 des PRV au Japon a une substitution d’acide aminé par rapport à celle des PRV en Europe, mais elle a montré une efficacité en tant que vaccin, conformément aux résultats précédents. Deg-CPR-1 a présenté une activité enzymatique de type cathepsine L. De plus, l’ARNm de Deg-CPR-1 était exprimé dans l’intestin moyen et à tous les stades où les PRV se nourrissent de sang. Ces résultats impliquent que Deg-CPR-1 dans l’intestin moyen peut avoir des fonctions importantes dans les processus physiologiques, et que l’inhibition de son expression peut contribuer à l’efficacité d’un vaccin basé sur Deg-CPR-1. Des recherches supplémentaires sont nécessaires pour comprendre pleinement les mécanismes de l’efficacité du vaccin.

## Introduction

The poultry red mite (PRM), *Dermanyssus gallinae* (De Geer 1778), one of the major haematophagous ectoparasites in poultry farming, causes severe economic loses, mainly to the laying hen sector [23]. Its infestations reduce egg production and quality, cause anaemia, and diminish disease resistance in chickens. Occasionally, severe infestations cause the death of juvenile chickens due to blood loss. Overall, the decreased productivity caused by PRM infestations creates serious problems for the poultry industry [23, 26]. In addition, PRMs may play a role as vectors in the transmission of avian pathogens [23]. Furthermore, PRM infestations often cause allergic reactions in humans, including both poultry workers and urban residents [1, 7, 13, 16], and a recent study reported the detection of zoonotic agents in PRMs [18]. Thus, PRMs are harmful pests to the poultry industry and a threat to human health.

Currently, PRMs are mainly controlled with pesticides; however, as they can hide in cracks, dust, and spider webs, PRM control with pesticides can be difficult to achieve. In addition, most pesticides offer only a limited or temporary reduction in PRM populations, and repeated or insufficient pesticide usage often results in the selection of pesticide-resistance properties in PRMs [23]. Generally, the pesticide-resistance properties vary with each farm, and this must be considered during pesticide selection. As a result, pesticide-resistant PRMs are difficult to control, and therefore, other management strategies are required to ensure animal welfare and to reduce the economic losses in poultry farming [23]. To date, several studies on new control strategies for PRMs have been published, including the following: development of repellents using plant oils [6, 22, 24], application of pathogens or predators of PRMs [15, 25], and conferring protective effects against PRMs in chickens by vaccination [2, 3, 4, 8, 28]. Of these, we have focused on vaccination as a protective strategy because its effects are expected to be prolonged if the antibody titre is induced to an adequate level and the influence of environmental factors can be reduced.

Cysteine proteases are crucial proteolytic enzymes for biological processes in various organisms from mammals to microbes and are involved in fundamental functions, such as catabolism and protein processing. In mammals, cysteine proteases are mainly localised to lysosomes and are well-known as cathepsins [27]; furthermore, 11 cysteine proteases have been predicted in humans, using bioinformatic analyses [19]. Cysteine proteases from some parasites are also well-characterised, and those of *Plasmodium falciparum* are known to be involved in haemoglobin degradation, parasite egress, and surface protein processing [27]. Cysteine proteases, which are expressed in the gut of flatworms and nematodes, putatively contribute as digestive enzymes to the degradation of host proteins to absorbable nutrients [5]. In addition, cysteine proteases are reportedly involved in haemoglobin digestion, such as cathepsins B, C, and L, in some ticks [21], and are considered candidates for the development of anti-tick vaccines [9, 20].

Protective antigens in PRMs that could potentially be utilised for PRM control have been reported [2, 3, 4, 8, 28]; among them are two orthologous cysteine proteases, Dg-CatL-1 [2] and Deg-CPR-1 [4]. The vaccine efficacy of both these cysteine proteases has been assessed; the results revealed a significant decrease in the survival rate of PRMs when they were fed heparinised blood of chickens immunised with Deg-CPR-1 [4]. These results suggest that Deg-CPR-1 could be developed as an anti-PRM vaccine. To further investigate the potential of Deg-CPR-1 as a vaccine antigen, in this study, we further characterised its genetic characteristics, enzyme activity, and gene expression.

## Materials and methods

### Sample collection

Poultry red mites (PRMs) were collected into 50 mL bioreactor tubes with vent caps (Corning Inc., Corning, NY, USA) from several poultry farms in Japan, transferred to the laboratory, and then maintained in an incubator at 25 °C under dark until use. A portion of the PRMs was designated as “fed PRMs”, and within 48 h of collection they were placed in 70% ethanol. The remaining PRMs, designated as “starved PRMs” were maintained at 25 °C for 7 weeks, and then placed in 70% ethanol. A portion of the starved PRMs was sorted according to their different life stages, namely protonymphs, deutonymphs, and adults, under a stereomicroscope SZX10 (Olympus, Tokyo, Japan), based on size and morphology. The fed PRMs, starved PRMs, and sorted PRMs were used in the expression analysis.

### RNA preparation and cDNA synthesis

The total RNA from each mite sample described above was extracted using the TRIzol reagent (Invitrogen, Carlsbad, CA, USA), according to the manufacturer’s protocol. One microgram of the total RNA was treated with DNase I (Invitrogen) to remove residual DNA, and cDNA was synthesised from the total RNA with PrimeScript Reverse Transcriptase (Takara Bio Inc., Shiga, Japan) using 200 pmol of oligo (dT)18 primer or 300 pmol of random hexamer primer (Hokkaido System Science, Hokkaido, Japan).

### Phylogenetic analysis

To analyse the genetic characteristics, the cysteine protease genes of two PRMs, one prevalent in Europe and one in Japan, were subjected to phylogenetic analysis (European: Deg-CPR-1 (EU), accession No.: KR697573; Japanese: Deg-CPR-1(Japan), accession no.: HZ459284). The phylogenetic analysis was performed using the cysteine protease genes of arthropods, including other mites and ticks, chickens, and other species, such as insects and invertebrates (Supplementary Table 1). The sequences were aligned using MEGA X software [14]. Thereafter, a maximum-likelihood phylogenetic tree was constructed using the same software with 1000 bootstrap replicates, with a JTT matrix-based model [11] using discrete Gamma distribution (+G) and by assuming that a certain fraction of sites is evolutionarily invariable (+I), to improve the tree topology.

### Expression of recombinant proteins

For immunisation, Deg-CPR-1 was prepared as a recombinant protein. The predicted coding region of Deg-CPR-1 at positions 19–549 (without the signal peptide amplified by PCR with z-Taq polymerase [Takara Bio Inc.]) (Supplementary Fig. 1B) was amplified using the following primers with NdeI and BamHI sites: 5′ – AGG TAG GCA TAT GGT TTC GGT GCC CCG AGG – 3′ (the underlined sequence indicates the NdeI site) and 5′ – CGC GGA TCC CTA CAG CTC GAC GTA GGT TG – 3′ (the underlined sequence indicates the BamHI site). The amplified fragments were cloned into the pET-19b vector (Merck, Darmstadt, Germany). The constructs obtained were transformed into *Escherichia coli* strain Rosetta-gami B (DE3) pLysS (Merck). The expression and purification of the recombinant protein were carried out per the manufacturer’s instructions. The recombinant Deg-CPR-1 was extracted from the insoluble inclusion body using the BugBuster^®^ Protein Extraction Reagent (Merck) and solubilised in 50 mM CAPS (pH 11) containing 0.3% N-lauroylsarcosine. The recombinant proteins were purified using TALON Metal Affinity Resins (Takara Bio Inc.). The eluted fractions were dialysed with 10 mM Tris-HCl (pH 8.5) containing 0.1 mM DTT to enable refolding of the recombinant protein. The concentration of the recombinant proteins was determined using the DC protein assay (Bio-Rad, Hercules, CA, USA).

To analyse the enzyme activity of Deg-CPR-1, the coding region of Deg-CPR-1 without the signal peptide at positions 19–549 (Supplementary Fig. 1B) was amplified using the following primers with NdeI and XbaI sites: 5′ – AGG TAG GCA TAT GGT TTC GGT GCC CCG AGG – 3′ (the underlined sequence indicates the NdeI site) and 5′ – CCT ATC TAG ACT ACA GCT CGA CGT AGG TTG – 3′ (the underlined sequence indicates the XbaI site). The peptidase domain (PD) of Deg-CPR-1 was present at positions 335–547 (Supplementary Fig. 1B), and therefore, the region including the PD, at positions 332–549, was amplified by PCR using the following primer set: 5′ – GAG GCA TAT GCG CGT CGA GCC GGA CTA CGT – 3′ (the underlined sequence indicates the NdeI site) and 5′ – CCT ATC TAG ACT ACA GCT CGA CGT AGG TTG – 3′ (the underlined sequence indicates the XbaI site). The amplified fragments were then cloned into the pCold-TF vector (Takara Bio Inc.). The constructs were transformed into *E. coli* strain Rosetta-gami B (DE3) pLysS (Merck), and the recombinant proteins were prepared as instructed by the manufacturer. The PD and the coding region without the signal peptide were expressed as a recombinant protein fused with a histidine-tagged trigger factor (TF), which contributes to the enhanced solubility of the fusion partners. The PD and Deg-CPR-1 were extracted from the soluble fraction and insoluble inclusion body, respectively, using the BugBuster Protein Extraction Reagent (Merck) and solubilised in 50 mM CAPS (pH 11) containing 0.3% N-lauroylsarcosine. The recombinant proteins were purified using TALON Metal Affinity Resins (Takara Bio Inc.) The eluted fractions were dialysed with 10 mM Tris-HCl (pH 8.5) containing 0.1 mM DTT to refold the recombinant protein. The concentration of the recombinant proteins was determined using the BCA protein assay reagent (Thermo Fisher Scientific, Waltham, MA, USA). The recombinant proteins of the PD and the whole coding region of Deg-CPR-1 without signal peptides were named as Deg-CPR-1(PD)-TF and Deg-CPR-1(whole)-TF, respectively. The concentration of the proteins was determined using the Pierce™ BCA Protein Assay Kit (Thermo Fisher Scientific), according to the manufacturer’s instructions.

To confirm protein purification, the obtained proteins were lysed in 2× SDS buffer (125 mM Tris–HCl (pH 6.8), 4% sodium dodecyl sulphate (SDS), 10% 2-mercaptoethanol, and 20% glycerol), boiled for 5 min, separated by 12% SDS-polyacrylamide gel electrophoresis (SDS-PAGE), and stained with Coomassie brilliant blue (FUJIFILM Wako Pure Chemical Corporation, Osaka, Japan).

### Immunisation with Deg-CPR-1

Animal experiments were conducted according to relevant guidelines and regulations of Choka Research Institute, Vaxxinova Japan K.K. (Tochigi, Japan), and the protocol was approved by the Animal Care and Use Committee, Vaxxinova Japan K.K. (Approval number: AR007-C-EX-015). Four chickens were intra-muscularly immunised with 25 μg of Deg-CPR-1 at 12 weeks of age. The recombinant Deg-CPR-1 was diluted in PBS and mixed with light liquid paraffin as the adjuvant. Ten weeks later, 25 μg of Deg-CPR-1 with the same adjuvant was used for a second round of immunisation. The plasma was isolated from the heparinised blood of the immunised chickens 5 weeks after the second immunisation. The plasma was also isolated from the heparinised blood of three unimmunised chickens of the same age. The obtained plasma was utilised in the *in vitro* feeding assay. All chickens were raised in the animal facility at Choka Research Institute, vaxxinova Japan K.K.

### Western blotting

The production of specific antibodies against Dg-Cys-his was examined by western blotting. Purified Dg-Cys-his was separated using a 12% SDS-polyacrylamide gel, and then transferred on to polyvinylidene difluoride membranes (Merck Millipore, Burlington, MA, USA). The membranes were blocked overnight with 0.05% Tween 20 in phosphate-buffered saline (PBST) containing 1% skim milk at 4 °C. The membranes were incubated at 25 °C with the isolated plasma from immunized chickens, washed three times with PBST, and incubated at 25 °C with anti-chicken IgY peroxidase rabbit antibody (Sigma-Aldrich). Finally, the membranes were incubated with Immobilon Western Chemiluminescent HRP Substrate (Merck Millipore) to visualise the peroxidase signal.

### Enzyme-linked immunosorbent assay

Antibody titres in the plasmas were determined by enzyme-linked immunosorbent assay **(**ELISA). The recombinant Deg-CPR-1 was coated on the wells of 96-well plates (Thermo Fisher Scientific) (125 ng/well) at 4 °C overnight with carbon-bicarbonate buffer. After washing each well three times with PBS, 10% BSA Diluent/Blocking Solution (SeraCare Life Sciences, Milford, MA, USA) was diluted 10-fold with PBS, and the blocking solution was added into each well; then, the plates were incubated at 25 °C for 16 h. After blocking, Wash Solution Concentrate (20×) (SeraCare Life Sciences) was diluted 20-fold, and each well was washed three times with this solution. After diluting the plasma 500-fold with the dilution solution (15-fold diluted 10% BSA Diluent/Blocking Solution [SeraCare Life Sciences]), the plasma was serially diluted to 32,000-fold. The diluted plasma samples were added into each well, and then the plates were incubated at 37 °C. After incubation for 2 h, the wells were washed three times with the same wash solution described above, and incubated at 37 °C with anti-chicken IgY[IgG](H + L)-HRP, Goat (Bethyl laboratories, TX, USA) for 1 h. As a substrate, 100 μL of SureBlue™ TMB Microwell Peroxidase Substrate (1-component) (SeraCare Life Sciences) was added into each well, and the plate was incubated at 37 °C for 15 min. After adding 100 μL of TMB Stop Solution (SeraCare Life Sciences) into each well, the absorbance of the sample was measured at 450 nm. The plasma from unimmunised chickens was diluted 500-fold, and its absorbance was measured in the same manner. The cut-off value was set to three times the values of the plasma, which was diluted 500-fold, from unimmunised chickens. The antibody titre was indicated as the maximum dilution rate.

### *In vitro* feeding assay

The devices for the *in vitro* feeding assay were prepared as previously reported [4]. For blood feeding the PRMs, the plasma obtained from fresh and heparinised chicken blood was replaced with the plasma obtained from immunised or unimmunised chicken blood. The plasma samples isolated from four immunised and three unimmunised chickens were tested. Blood feeding was performed at 40 °C overnight, and approximately 20 PRMs were collected in Pasteur pipettes; only blood-feeding PRMs were collected. The mortality of blood-feeding PRMs was monitored every day for 1 week. The total number of blood-feeding PRMs monitored was as follows: Deg-CPR-1: total, *n* = 155 (in each plasma sample *n* = 42, 35, 32, and 46); Control: total, *n* = 89 (*n* = 18, 24, and 47).

### Enzyme activity of the Deg-CPR-1 proteins

Deg-CPR-1(PD)-TF and Deg-CPR-1(whole)-TF were used for the enzyme activity assay. TF alone was used as the negative control. Their enzyme activity was assessed using the SensoLyte Rh110 Cathepsin L Assay Kit (AnaSpec, Inc., Fremont, CA, USA), according to the manufacturer’s instructions. Fluorescence was detected at excitation/emission wavelengths of 490/530 nm. The enzyme activity was assessed using 10, 50, 100, and 200 μg/mL recombinant proteins.

### Laser-capture microdissection and cDNA synthesis

The starved PRMs were fixed with 4% paraformaldehyde and embedded in paraffin for laser-capture microdissection (LCM). LCM was performed as previously reported [10]. First, 5-μm-thick paraffin sections were mounted on glass slides precoated with LCM films (Meiwafosis, Tokyo, Japan), deparaffinised with xylene, and dehydrated with alcohol. After staining with 1% toluidine blue for 5 s, LCM of the salivary gland, midgut, and ovary was performed using Ls-Pro300 (Meiwafosis), according to the manufacturer’s protocol. All procedures were performed in RNase-free conditions. The total RNA was purified using the RNAqueous Micro Total RNA Isolation kit (Thermo Fisher Scientific) and was reverse-transcribed to cDNA using the SuperScript First-Strand Synthesis System for RT-PCR (Thermo Fisher Scientific), according to the manufacturer’s protocol.

### Expression analysis of Deg-CPR-1

The expression of *Deg-CPR-1* mRNA in the fed and starved PRMs, and in different life stages, protonymphs, deutonymphs, and adults, was analysed using standard PCR protocols. For the expression analysis of *Deg-CPR-1* in the fed and starved PRMs, we used samples from two different farms. The PCR was performed using Ex-Taq polymerase (Takara Bio Inc.) according to the manufacturer’s protocol, and the primers used were as follows: forward, 5′ – GTC TGG ACT TTA TCG CCT ACC – 3′ and reverse, 5′ – TTC GGT AGC CTT TCA GCG TGC – 3′. We also used the primers for the *actin* gene as an internal control, and their sequences were as follows: forward, 5′ – CCC GAC GGA CAG GTG ATT ACC – 3′ and reverse, 5′ – TCG AGC CTC CGA TCC AGA CGG – 3′. The expression of *Deg-CPR-1* mRNA in the tissue samples collected by LCM was analysed by nested PCR. The first round of PCR was performed as described above. After amplification, 1 μL of the reaction mixture was used for the second round of PCR, and it was performed using the following primer sets: Deg-CPR-1 forward, 5′ – CGC CTA CCA CTA CAT CCA GAA – 3′ and reverse, 5′ – GCT AAT GGG CTT GTT GGA AA – 3′; actin forward, 5′ – TGA TTA CCA TTG GCA ACG AG – 3′ and reverse, 5′ – GTG TTG GCG TAC AGG TCC TT – 3′. The amplified products were subjected to electrophoresis on a 2.0% agarose gel.

### Statistical analysis

To compare the mortality of PRMs between the immunised and control groups after *in vitro* feeding, Kaplan–Meier curves were generated and a log-rank test was performed. In addition, the mortality of PRMs on each day throughout the experimental period was compared between the immunised and control groups using the chi-squared test, and the odds ratio and 95% confidence interval (CI) were estimated. In the enzyme activity assay, all values are expressed as mean ± standard deviation, and a statistical comparison was performed using a one-way analysis of variance, with a Tukey HSD test for post hoc comparisons. All statistical analyses were performed using EZR [12], and results with *p* < 0.01 were considered statistically significant.

## Results

### Phylogenetic analysis of *Deg-CPR-1*

We cloned and determined the nucleotide sequence of *Deg-CPR-1* of PRMs collected from poultry farms in Japan (Deg-CPR-1 (Japan), accession no.: HZ459284) (Supplementary Table 1). A comparison of this nucleotide sequence with that of European PRMs (Deg-CPR-1 (EU), accession no.: KR697573) (Supplementary Table 1) revealed several differences. While most of the differences were synonymous substitutions, an amino acid substitution was found at position 535 (aspartic acid in PRMs in Japan; asparagine in PRMs in Europe) when the deduced amino acid sequences were compared (Supplementary Fig. 1A). According to the BLAST search, Deg-CPR-1 was predicted as a cysteine protease that has a cathepsin propeptide inhibitor domain at positions 249–305 and a peptidase C1A domain at positions 335–547 (Supplementary Figs. 1A and 1B). In addition, an amino acid substitution at position 535 was not observed in the amino acid residues predicted as the active sites for peptidase, and it does not seem to affect the enzymatic activity. Therefore, Deg-CPR-1 was predicted as a cysteine protease that exhibits enzyme activity by the cleavage of the inhibitor domain.

**Figure 1:**
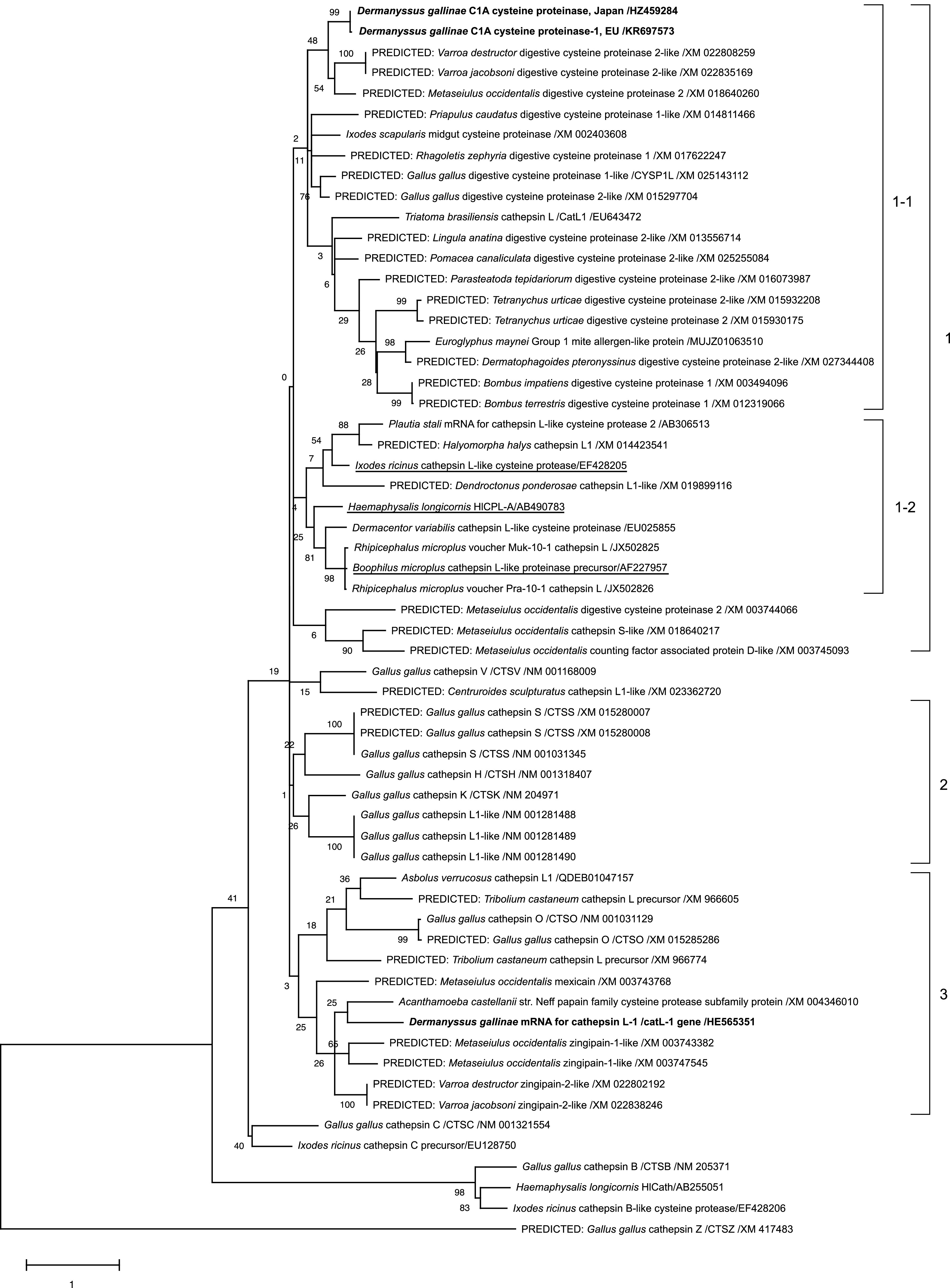
Phylogenetic tree based on the nucleotide sequences of the open reading frames of the *cysteine protease* genes in poultry red mites (PRMs, *Dermanyssus gallinae*), arthropods, including other mites and ticks, chickens, and other species such as insects and invertebrates. The tree was constructed using the maximum-likelihood method with MEGA X software [14]. The numbers on the right indicate the clusters. Cluster 1: This cluster was divided into two sub-clusters. The digestive cysteine proteases from various species were classified into cluster 1–1, and cysteine proteases from PRMs (*Deg-CPR-1*) belonged to this cluster (bold font). Sub-cluster 1–2 included cathepsin L-like proteases and some cysteine proteases that are proposed to play a role in haemoglobin digestion in ticks (underlined). Cluster 2: This cluster comprised the cysteine proteases and cathepsins S, H, K, and L of chickens. Cluster 3: The cysteine proteases from mites, different from those in cluster 1 were included in this cluster, and another cysteine protease from PRMs, Dg-CatL-1, was also included (bold font).

To compare the genetic characteristics of *Deg-CPR-1* (Japan) and *Deg-CPR-1* (EU) with another cysteine protease of PRMs, *Dg-CatL-1*, and various cysteine proteases from arthropods, chickens, and other species, such as insects and invertebrates, phylogenetic analysis was performed (Fig. 1). The *Deg-CPR-1* genes belonged to cluster 1, and they were closely related to the cysteine proteases from other mite species, such as predatory mites and varroa mites (Fig. 1). In addition, cluster 1 was divided into two sub-clusters, and the *Deg-CPR-1* genes belonged to cluster 1–1 that included the digestive cysteine proteases of various species, including chickens. In another sub-cluster (cluster 1–2), cathepsin L-like proteases were included. Among them, some cathepsin L-like proteases identified in ticks were included, and previous reports suggest that these cathepsin L-proteases contributed to haemoglobin digestion [21]. In contrast, *Dg-CatL-1* was classified into a different cluster (cluster 3) from cluster 1, and cysteine proteases of mites, different from those in cluster 1, mainly belonged to this cluster. The cysteine proteases, cathepsins S, H, K, and L, of chickens formed an independent cluster (cluster 2) different from the cysteine proteases of other species. Thus, the phylogeny of *Deg-CPR-1* appears to be close to that of digestive cysteine proteases broadly encoded in various species, and clearly different from that of *Dg-CatL-1* and chicken cathepsins.

### Assessment of the acaricidal activity of plasma from chickens immunised with Deg-CPR-1

**Figure 2:**
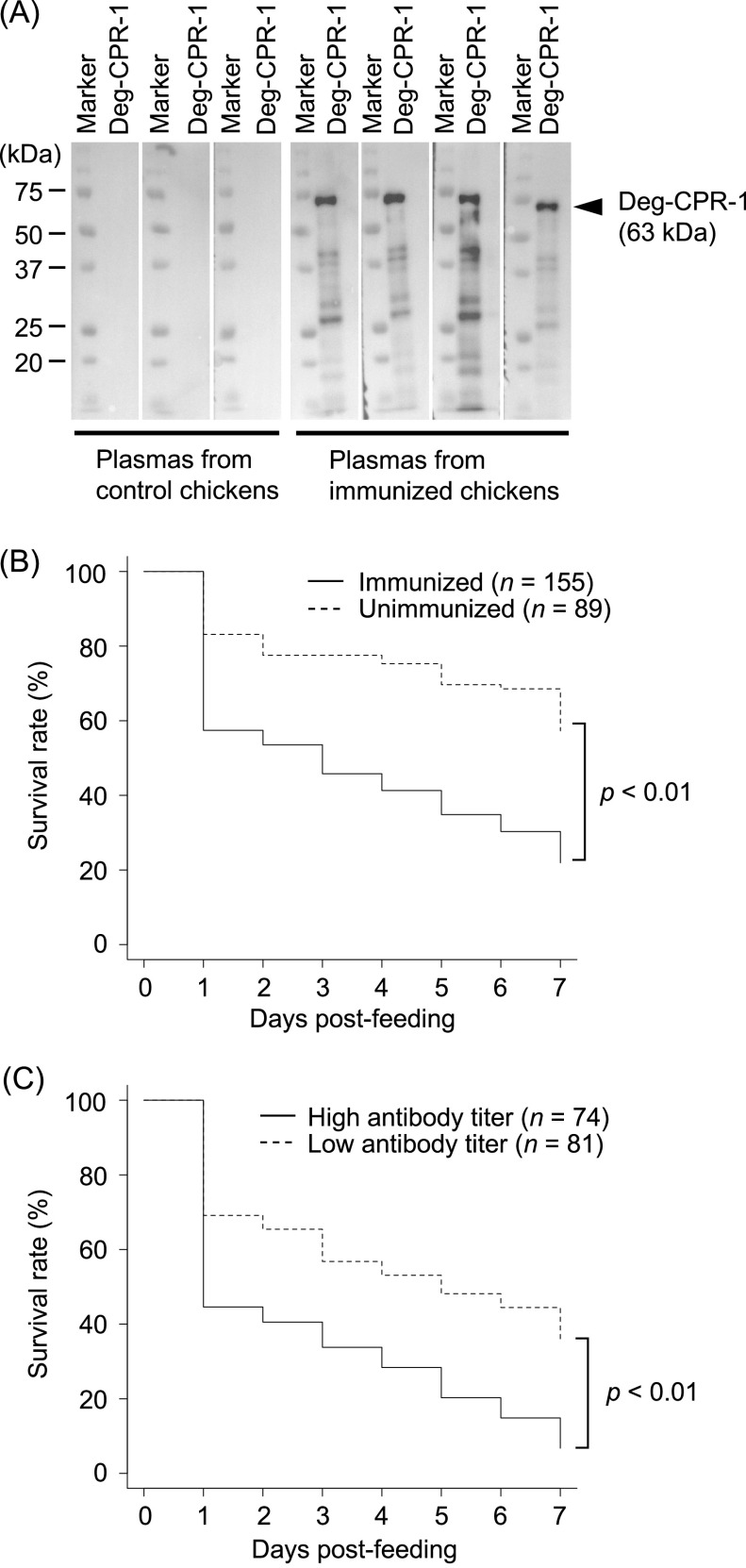
Assessment of the acaricidal potential of the plasma from chickens immunised with the recombinant cysteine protease protein from PRMs (Deg-CPR-1) against poultry red mites (PRMs, *Dermanyssus gallinae*). (A) The production of antibody in the plasma from chickens immunised with Deg-CPR-1. Four chickens were immunised with Deg-CPR-1 and three chickens were unimmunised. The plasma was isolated from immunised and unimmunised chickens, and the antibody specific to Deg-CPR-1 in the plasma was detected by western blotting. The arrow head indicates the predicted molecular weight of Deg-CPR-1 (63 kDa). (B and C) The mortality rate of PRMs that were fed blood containing the plasma from immunised chickens was assessed every day for 1 week. Four and three plasma samples isolated from the immunised or unimmunised chickens were used in the *in vitro* feeding assays, and the total number of blood-feeding PRMs monitored was as follows: immunised: *n* = 155; unimmunised: *n* = 89 (B). The total number of PRMs fed blood containing the plasma with high antibody titre or low antibody titre was as follows: high antibody titre: *n* = 74; low antibody titre: *n* = 81 (C). The number of dead PRMs was recorded and plotted on the graph to generate Kaplan–Meier curves. Statistical analysis was performed using a log-rank test. Results with *p* < 0.01 were considered statistically significant.

A previous report revealed a significant increase in the mortality of PRMs that fed on the heparinised blood of chickens immunised with Deg-CPR-1, compared with that of PRMs fed the blood of chickens immunised with adjuvant only [4]. To confirm the acaricidal potential of immunisation with Deg-CPR-1 from Japan, we briefly compared the mortality rate of PRMs fed the plasma of immunised and unimmunised chickens, using an *in vitro* feeding assay. The recombinant Deg-CPR-1 was expressed and purified (Supplementary Fig. 2). We immunised the chickens with recombinant Deg-CPR-1 and obtained the plasma. The production of antibodies specific to Deg-CPR-1 was confirmed by western blotting using the immunised-chicken plasma (Fig. 2A). The mortality of PRMs fed the immunised-chicken plasma was monitored for 7 days post-feeding. To evaluate the acaricidal potential of Deg-CPR-1 immunisation, we statistically analysed the difference in the mortality rate between the unimmunised and immunised groups using the chi-squares test at each time point and log-rank test. The mortality of PRMs fed plasma from immunised chickens increased throughout the observation period, compared with that of the unimmunised group (Table 1). In addition, the odds ratio indicated increased mortality of PRMs at all time points. A comparison of the Kaplan–Meier curves revealed that the mortality of PRMs fed the plasma from immunised chickens was significantly increased compared with that of PRMs fed the plasma from unimmunised chickens (Fig. 2B). Thus, the acaricidal potential of immunisation with Deg-CPR-1 from Japan was confirmed, and this is consistent with the findings of a previous study [4]. In addition, we analysed the antibody titre in the plasma obtained from immunised chickens. The antibody titre increased in the plasma of all chickens, and especially, chickens A and C presented higher antibody titres (Table 2). Therefore, we compared the mortality of PRMs fed plasma with higher and lower antibody titres. The mortality of PRMs fed the plasma with higher antibody titre was higher than that of PRMs fed plasma with lower antibody titres throughout the observation period, and the odds ratio also indicated the increased mortality at all time points (Table 3). The Kaplan–Meier curves revealed that the mortality of PRMs fed plasma with higher antibody titre was significantly increased (Fig. 2C). Thus, immunisation with Deg-CPR-1 has potential acaricidal activity, and the efficacy seems to depend on the immunological status of the immunised chickens.

**Table 1:** Mortality of PRMs fed the plasma of chickens immunised with Deg-CPR-1.

	Days post-feeding						
	1	2	3	4	5	6	7
Immunised group (*n* = 155)							
No. of dead PRMs post-feeding	66	72	84	91	101	108	121
Mortality (%)	42.58	46.45	54.19	58.71	65.16	69.68	78.06
Unimmunised group (*n* = 89)							
No. of dead PRMs post-feeding	15	20	20	22	27	28	38
Mortality (%)	16.85	22.47	22.47	24.72	30.34	31.46	42.70
Chi-square	16.87	13.84	23.26	26.27	27.49	33.47	31.15
*p* value	<0.01	<0.01	<0.01	<0.01	<0.01	<0.01	<0.01
Odds ratio	3.66	2.99	4.08	4.33	4.29	5.01	4.78
95% CI (lower limit)	1.97	1.68	2.30	2.47	2.49	2.90	2.76
95% CI (upper limit)	6.79	5.33	7.23	7.58	7.41	8.64	8.27

**Table 2:** Antibody titre against Deg-CPR-1 in the plasma of immunised chickens.

Immunised chicken	Antibody titre
A	32,000
B	16,000
C	64,000
D	16,000

**Table 3:** Comparison of the mortality rate of PRMs fed the plasma with higher and lower titres of antibodies against Deg-CPR-1.

	Days post-feeding						
	1	2	3	4	5	6	7
Immunised chickens with high antibody titre (*n* = 74)							
No. of dead PRMs post-feeding	41	44	49	53	59	63	69
Mortality (%)	55.41	59.46	66.22	71.62	79.73	85.14	93.24
Immunised chickens with low antibody titre (*n* = 81)							
No. of dead PRMs post-feeding	25	28	35	38	42	45	52
Mortality (%)	30.86	34.57	43.21	46.91	51.85	55.56	64.20
Chi-square	9.53	9.63	8.25	9.74	13.24	16.01	19.05
*p* value	<0.01	<0.01	<0.01	<0.01	<0.01	<0.01	<0.01
Odds ratio	2.78	2.78	2.58	2.86	3.65	4.58	7.70
95% CI (lower limit)	1.45	1.46	1.35	1.48	1.82	2.17	3.08
95% CI (upper limit)	5.33	5.29	4.91	5.52	7.34	9.66	19.24

### Enzyme activity of Deg-CPR-1

**Figure 3:**
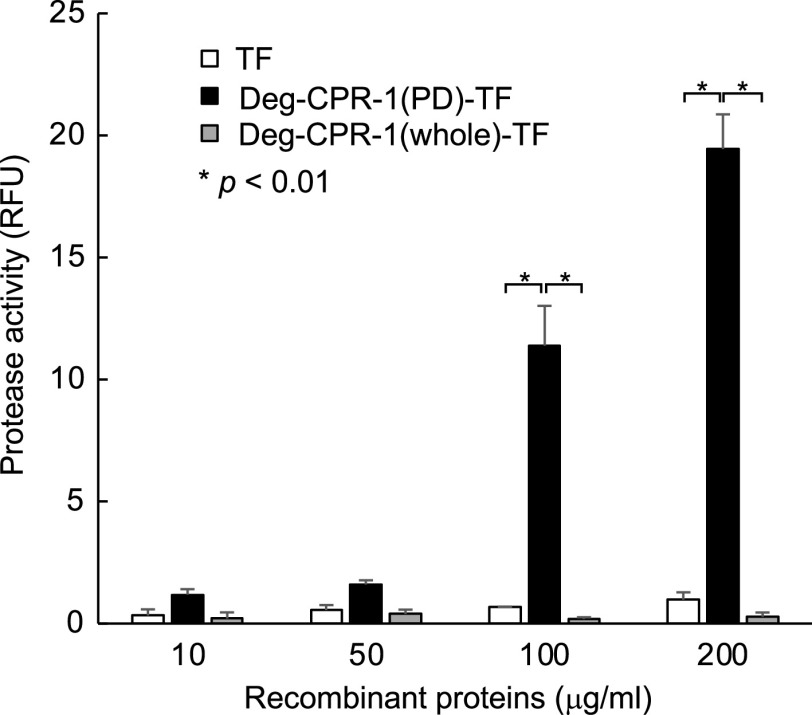
Enzyme activity of the recombinant cysteine protease proteins from poultry red mites (PRMs, *Dermanyssus gallinae*) (Deg-CPR-1). The enzyme activity of the peptidase domain of Deg-CPR-1 (Deg-CPR-1(PD)-TF) and the whole region of Deg-CPR-1 without signal peptides (Deg-CPR-1(whole)-TF), including the inhibitor domain, was assessed using a SensoLyte Rh110 Cathepsin L Assay Kit (AnaSpec, Inc., Fremont, CA, USA) to measure cathepsin L activity. The concentrations of recombinant proteins used in each assay are indicated along the *x*-axis. TF alone was used as a negative control. Protease activity was indicated as relative fluorescence units (RFU). Error bars indicate standard deviations. Results with *p* < 0.01 were considered significant.

To investigate the cathepsin L-like protease functions of Deg-CPR-1, we tested the enzyme activity using a commercial kit for the cathepsin L assay. It was predicted that Deg-CPR-1 has a PD at the C-terminus and a peptidase inhibitor domain in the upstream region of the PD (Supplementary Fig. 1A). In contrast, no domain was found in the region at the N-terminus of Deg-CPR-1. Therefore, we expressed the C-terminus region, including the PD (Deg-CPR-1(PD)-TF), without the inhibitor domain, and the entire region without signal peptides (Deg-CPR-1(whole)-TF) as recombinant proteins fused with TF (Supplementary Fig. 1B). Deg-CPR-1(PD)-TF and Deg-CPR-1(whole)-TF were purified from the soluble and insoluble fractions, respectively (Supplementary Fig. 3), and were subjected to the enzyme activity assay. Deg-CPR-1(PD)-TF showed a dose-dependent cathepsin L-like enzyme activity, whereas Deg-CPR-1(whole)-TF and TF exhibited no enzyme activity (Fig. 3). These results suggest that Deg-CPR-1 potentially has cathepsin L-like enzyme activity.

### Expression analysis of Deg-CPR-1

**Figure 4:**
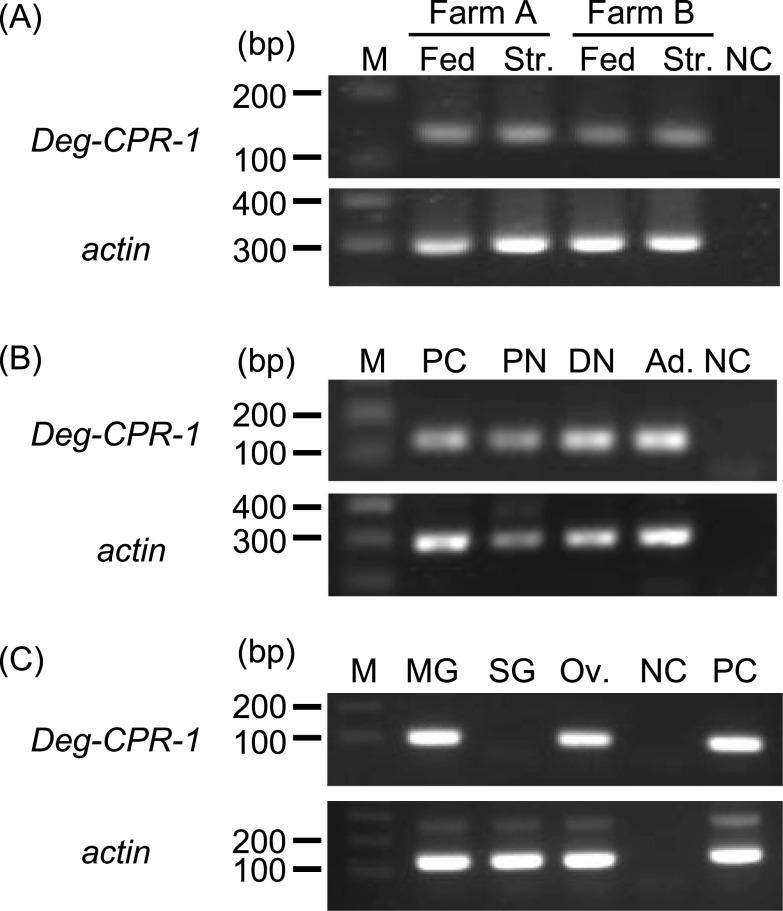
Expression analysis of the cysteine protease gene of poultry red mites (PRMs, *Dermanyssus gallinae*) (*Deg-CPR-1*). (A) Expression analysis of *Deg-CPR-1* in fed and starved PRMs. Some PRMs were fixed after their transfer to the laboratory, designated as “fed PRMs”, and the remaining PRMs were maintained at 25 °C for 7 weeks to digest the blood they had ingested; these were designated as “starved PRMs”. The *Deg-CPR-1* mRNA was detected in PRMs from two different farms by RT-PCR. Fed: fed PRMs, Str.: starved PRMs, NC: negative control (distilled water). (B) Expression analysis of *Deg-CPR-1* in PRMs fed blood at different life stages. A portion of the starved PRMs were sorted according to their life stages, namely, protonymphs, deutonymphs, and adults, under a stereomicroscope, based on their morphology and body size, and *Deg-CPR-1* mRNA was detected by RT-PCR. PC: positive control (cDNA from PRMs of all stages), PN: protonymph, DN: deutonymph, Ad: adult, NC: negative control (distilled water). (C) Expression analysis of *Deg-CPR-1* in different tissues. The tissue samples were collected from starved deutonymphs and adults by laser-capture microdissection, and RT – nested PCR was performed to detect the *Deg-CPR-1* mRNA in the midgut, salivary gland, and ovary. PC: positive control (cDNA from PRMs of all stages), MG: midgut, SG: salivary gland, Ov: ovary, NC: negative control (distilled water). The *actin* gene was amplified as an internal control in all expression analyses.

To further characterise Deg-CPR-1, we performed an expression analysis of *Deg-CPR-1* mRNA. First, we analysed the expression in fed or starved PRMs in mixed stages to identify whether the expression was dependent on blood feeding. The expression of *Deg-CPR-1* was observed in PRM samples from two independent farms, regardless of blood feeding (Fig. 4A). Next, we investigated the expression in different life stages during which PRMs feed on blood; *Deg-CPR-1* mRNA was detected during the protonymph, deutonymph, and adult stages, as the PRMs start to feed on blood from the protonymph stage. The *Deg-CPR-1* mRNA was detected in all stages and analysed (Fig. 4B). Finally, we examined the expression sites of *Deg-CPR-1* mRNA using LCM and RT-nested PCR. For these analyses, we used tissue samples from the midgut, salivary gland, and ovary. Expression of *Deg-CPR-1* was observed in the midgut and ovary (Fig. 4C), suggesting that it might be constitutively expressed in the midgut and ovary of PRMs, regardless of blood feeding.

## Discussion

In the present study, we investigated the characteristics of Deg-CPR-1, a cysteine protease, from PRMs. An amino acid substitution was observed within Deg-CPR-1 when European and Japanese PRMs were compared. However, the position of amino acid substitution was different from the active sites for cysteine proteases, and therefore, this substitution does not seem to affect the enzyme activity. The phylogenetic analysis showed that *Deg-CPR-1* was closely related to genes encoding digestive cysteine proteases from other mite species, and it was classified into the same cluster as the cathepsin L-like proteases that are predicted to be enzymes related to haemoglobin digestion in the midgut of ticks [21]. The enzyme activity assay revealed that the PD of Deg-CPR-1 has the cathepsin L-like protease function. Furthermore, the expression of *Deg-CPR-1* was observed in both PRMs, fed and starved, at all blood-feeding life stages, and in the midgut. Taken together, these results suggest that Deg-CPR-1 is a cysteine protease that contributes to protein digestion in the midgut of PRMs and that this protein could be a suitable candidate for the development of anti-PRM vaccines.

Cysteine proteases consist of a prodomain and mature domain. Two highly conserved motifs, ERFNIN and GNFD, are present in the prodomains of the cathepsin L subfamily, whereas cathepsins B, C, O, and X lack the ERFNIN motif [27]. The ERFNIN and GNFD-like motifs were also found in Deg-CPR-1, and the amino acid residues of the motifs were ERFNVN and KNRD, respectively. Amino acid diversity in both motifs has been reported in falcipains, which are malarial cysteine proteases belonging to a cathepsin L-like subfamily [17]. We observed that the recombinant Deg-CPR-1 proteins exhibited cathepsin L-like enzyme activity. Taken together, Deg-CPR-1 can be classified into the cathepsin L subfamily of PRMs. However, in this study, we used the substrate in a commercial kit, which is used for measuring human and other mammalian cathepsin L activity. To further characterise the cathepsin L-like activity and the substrate specificity of Deg-CPR-1, the enzymatic activity should be analysed using several substrates for cathepsin L, as there may be a difference in substrate specificity between Deg-CPR-1 and cathepsins from human and other mammalian species.

Cathepsin L proteases are proposed to play a role in haemoglobin digestion in some tick species, such as *Rhipicephalus microplus*, *Haemaphysalis longicornis*, and *Ixodes ricinus* [21]. In addition, it has been reported that the enzymes related to haemoglobin digestion could be suitable candidates for the development of anti-tick vaccines [9, 20]. Bartley et al. first reported the potential of Deg-CPR-1 as a vaccine candidate [4], and in the present study, we observed the expression of *Deg-CPR-1* in the midgut of PRMs and a decrease in the survival of PRMs that were fed plasma from Deg-CPR-1-immunised chickens. Therefore, Deg-CPR-1 may contribute to haemoglobin digestion, and therefore, the acaricidal effect can be attributed to the inhibitory function. However, the expression of *Deg-CPR-1* was also confirmed in the ovary, suggesting different functions in other organs. In addition, *Deg-CPR-1* was classified into a sub-cluster different from that of cysteine proteases that were identified in ticks and related to haemoglobin digestion. Therefore, to confirm the roles of Deg-CPR-1, the effects of Deg-CPR-1 on haemoglobin digestion should be assessed. In addition, to investigate the importance of Deg-CPR-1 in the midgut and ovary, the effects of *Deg-CPR-1* silencing by RNA interference on the midgut and ovary should be evaluated. Furthermore, identifying other cysteine proteases, which are closely related to those identified from ticks, with a proposed role in haemoglobin digestion, may be necessary to develop better vaccines.

In this study, we characterised Deg-CPR-1, which is one of the vaccine candidates against PRMs. Deg-CPR-1 was predicted to be expressed in the midgut and during all blood-feeding life stages of PRMs, and this indicated cathepsin-L-like enzyme activity. In ticks, several proteases of reported biological importance have been identified, and therefore, they could be effective candidates as vaccine antigens [21]. To develop effective vaccines against PRMs, the effects of the vaccine have to be assessed using PRM-challenge trials in immunised chickens; in addition, the effects of various combinations of antigens as a cocktail vaccine should be assessed. Deg-CPR-1 could be a candidate for the development of such cocktail vaccines.

## Supplementary Material

Supplementary material is available at https://www.parasite-journal.org/10.1051/parasite/2021005/olm *Supplementary Table*. List of *cysteine protease* genes used for the phylogenetic analysis in Figure 1. *Supplementary Figure 1*. Structure of cysteine protease from poultry red mites (PRMs, *Dermanyssus gallinae*) (Deg-CPR-1) and recombinant proteins. (**A**) The nucleotide and amino acid sequences of Deg-CPR-1 from PRMs collected in Japan. Deg-CPR-1 has the signal peptides at positions 1–18 (dashed line), cathepsin propeptide inhibitor domain at positions 249–305 (grey box), and peptidase domain at positions 335–547 (white box). The black arrow-head indicates an amino acid difference in Deg-CPR-1 between Japanese PRMs and European PRMs at position 535 (aspartic acid in PRMs in Japan; asparagine in PRMs in Europe). The white arrow-heads indicate the predicted active sites for the catalytic residues of the peptidases. (**B**) The structure of Deg-CPR-1 in the PRMs and recombinant proteins used in this study. For immunisation, the entire recombinant proteins without signal peptides were fused with the histidine tag. For enzyme activity analysis, two recombinant Deg-CPR-1 proteins were used: The peptidase domain fused with histidine-tagged trigger factor (TF), and the whole region without signal peptides fused with histidine-tagged TF. The structure of recombinant protein used in the enzyme activity assay is indicated. *Supplementary Figure 2*. The expression and purification of cysteine protease from poultry red mites (PRMs, *Dermanyssus gallinae*) (Deg-CPR-1). The entire recombinant Deg-CPR-1 without the signal peptides, fused with the histidine tag, was expressed and purified for immunisation. The recombinant Deg-CPR-1 was purified from the insoluble inclusion body. The recombinant Deg-CPR-1 was extracted from the insoluble inclusion body and purified using the metal affinity resins. M: Marker (Precision Plus Protein™ All Blue Prestained Protein Standards, Bio-Rad, Hercules, CA, USA). *Supplementary Figure 3*. The expression and purification of cysteine protease from poultry red mites (PRMs, *Dermanyssus gallinae*) (Deg-CPR-1) fused with histidine-tagged trigger factor (TF). Two recombinant Deg-CPR-1 proteins fused with histidine-tagged TF were used. The peptidase domain fused with histidine-tagged TF (Deg-CPR-1(PD)-TF), the entire region without signal peptides fused with histidine-tagged TF (Deg-CPR-1(whole)-TF), and TF were expressed and purified for the analysis of enzyme activity. Deg-CPR-1(PD)-TF and TF were purified from the soluble fraction, and Deg-CPR-1(whole)-TF was purified from the insoluble inclusion body. The recombinant proteins were purified using the metal affinity resins. After the elution of recombinant proteins from the resins, the buffer was changed to phosphate-buffered saline by ultrafiltration. M: Marker (Precision Plus Protein™ All Blue Prestained Protein Standards, Bio-Rad).

## Conflict of interest

ES and AT are employed by Vaxxinova Japan K.K, Tokyo, Japan. SM, AT, MI, SK, and KO are authors of patent-covering materials and techniques described in this manuscript (EU patent, EP15800403.6; Japanese patent, 2016-523581). The other authors have no financial conflicts of interest.
