# Supplementary material for: Characterisation of a cysteine protease from poultry red mites and its potential use as a vaccine for chickens
Source: Parasite. 2021 Feb 3;28:9. doi: 10.1051/parasite/2021005 (PMC7863971; doi:10.1051/parasite/2021005)
Supplement: Supplementary file 1 — Supplementary Table. List of cysteine protease genes used for the phylogenetic analysis in Figure 1. [file parasite-28-9-s1.zip › parasite200142-1-olm/Tables_R1-2.pdf]

Table 1. Mortality of PRMs fed the plasma of chickens immunised with Deg-CPR-1

	Days post-feeding						
	1	2	3	4	5	6	7
Immunised group (*n* = 155)							
No. of dead PRMs post-feeding	66	72	84	91	101	108	121
Mortality (%)	42.58	46.45	54.19	58.71	65.16	69.68	78.06
Unimmunised group (*n* = 89)							
No. of dead PRMs post-feeding	15	20	20	22	27	28	38
Mortality (%)	16.85	22.47	22.47	24.72	30.34	31.46	42.70
Chi-square	16.87	13.84	23.26	26.27	27.49	33.47	31.15
P value	<0.01	<0.01	<0.01	<0.01	<0.01	<0.01	<0.01
Odds ratio	3.66	2.99	4.08	4.33	4.29	5.01	4.78
95% CI (lower limit)	1.97	1.68	2.30	2.47	2.49	2.90	2.76
95% CI (upper limit)	6.79	5.33	7.23	7.58	7.41	8.64	8.27

Table 2. Antibody titre against Deg-CPR-1 in the plasma of immunised chickens

Immunised chicken	Antibody titre
A	32,000
B	16,000
C	64,000
D	16,000

Table 3. Comparison of the mortality rate of PRMs fed the plasma with higher and lower titres of antibodies against Deg-CPR-1

	Days post-feeding						
	1	2	3	4	5	6	7
Immunised chickens with high antibody titre (*n* = 74)							
No. of dead PRMs post-feeding	41	44	49	53	59	63	69
Mortality (%)	55.41	59.46	66.22	71.62	79.73	85.14	93.24
Immunised chickens with low antibody titre (*n* = 81)							
No. of dead PRMs post-feeding	25	28	35	38	42	45	52
Mortality (%)	30.86	34.57	43.21	46.91	51.85	55.56	64.20
Chi-square	9.53	9.63	8.25	9.74	13.24	16.01	19.05
P value	<0.01	<0.01	<0.01	<0.01	<0.01	<0.01	<0.01
Odds ratio	2.78	2.78	2.58	2.86	3.65	4.58	7.70
95% CI (lower limit)	1.45	1.46	1.35	1.48	1.82	2.17	3.08
95% CI (upper limit)	5.33	5.29	4.91	5.52	7.34	9.66	19.24
