# Supplementary material for: Characterisation of a cysteine protease from poultry red mites and its potential use as a vaccine for chickens
Source: Parasite. 2021 Feb 3;28:9. doi: 10.1051/parasite/2021005 (PMC7863971; doi:10.1051/parasite/2021005)
Supplement: Supplementary file 2 — Supplementary Figure 1. Structure of cysteine protease from poultry red mites (PRMs, Dermanyssus gallinae) (Deg-CPR-1) and recombinant proteins. (A) The nucleotide and amino acid sequences of Deg-CPR-1 from PRMs collected in Japan. Deg-CPR-1 has the signal peptides at positions 1–18 (dashed line), cathepsin propeptide inhibitor domain at positions 249–305 (grey box), and peptidase domain at positions 335–547 (white box). The black arrow-head indicates an amino acid difference in Deg-CPR-1 between Japanese PRMs and European PRMs at position 535 (aspartic acid in PRMs in Japan; asparagine in PRMs in Europe). The white arrow-heads indicate the predicted active sites for the catalytic residues of the peptidases. (B) The structure of Deg-CPR-1 in the PRMs and recombinant proteins used in this study. For immunisation, the entire recombinant proteins without signal peptides were fused with the histidine tag. For enzyme activity analysis, two recombinant Deg-CPR-1 proteins were used: The peptidase domain fused with histidine-tagged trigger factor (TF), and the whole region without signal peptides fused with histidine-tagged TF. The structure of recombinant protein used in the enzyme activity assay is indicated. [file parasite-28-9-s1.pdf]

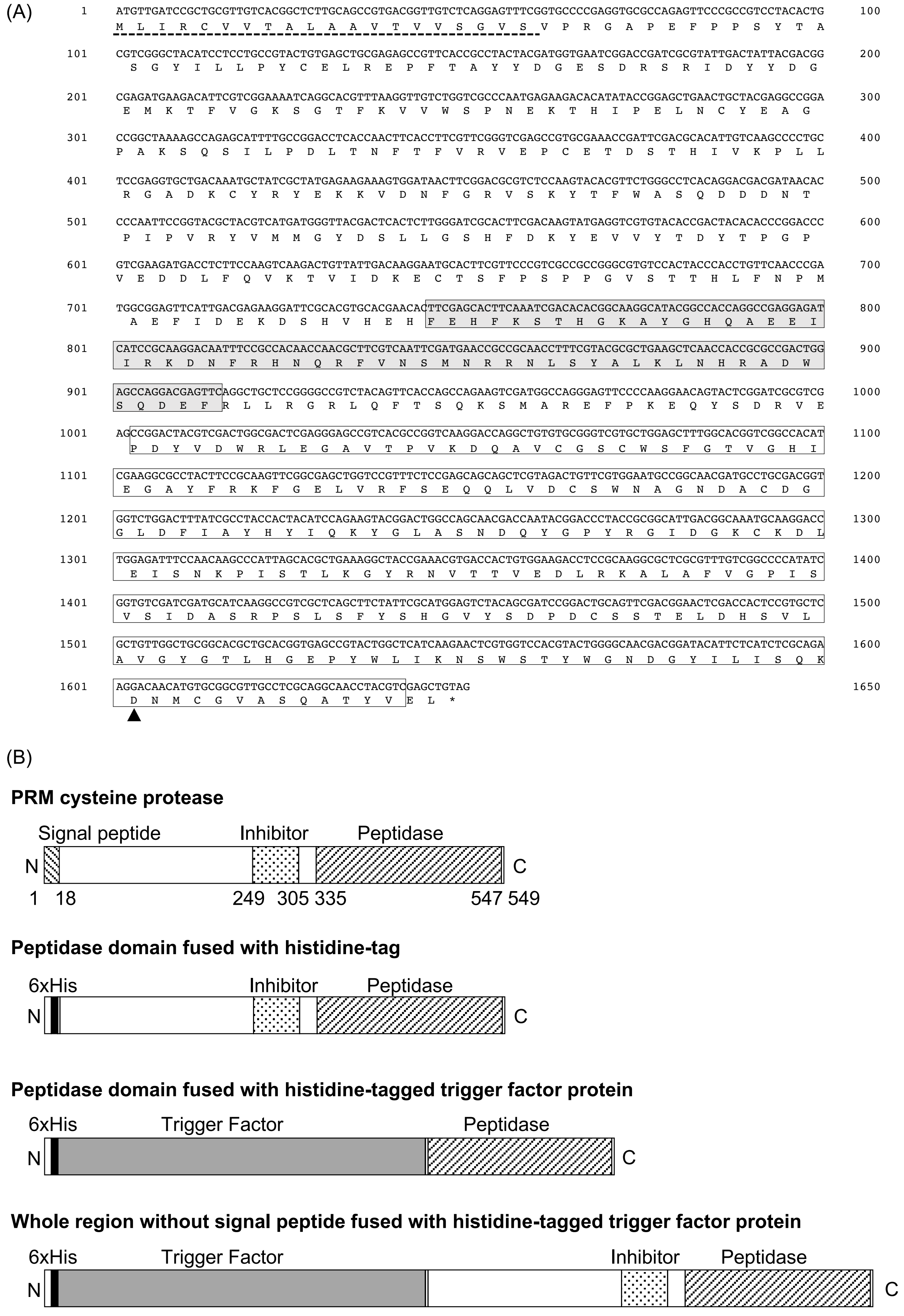
(A)
1 ATGTTGATCCGCTGCGTTGTCACGGCTCTTGCAGCCGTGACGGTTGTCTCAGGAGTTTCGGTGCCCCGAGGTGCGCCAGAGTTCCCGCCGTCCTACACTG 100
M L I R C V V T A L A A V T V V S G V S V P R G A P E F P P S Y T A
101 CGTCGGGCTACATCCTCCTGCCGTACTGTGAGCTGCGAGAGCCGTTCACCGCCTACTACGATGGTGAATCGGACCGATCGCGTATTGACTATTACGACGG 200
S G Y I L L P Y C E L R E P F T A Y Y D G E S D R S R I D Y Y D G
201 CGAGATGAAGACATTCGTCGGAAAATCAGGCACGTTTAAGGTTGTCTGGTCGCCCAATGAGAAGACACATATACCGGAGCTGAACTGCTACGAGGCCGGA 300
E M K T F V G K S G T F K V V W S P N E K T H I P E L N C Y E A G
301 CCGGCTAAAAGCCAGAGCATTTTGCCGGACCTCACCAACTTCACCTTCGTTCGGGTCGAGCCGTGCGAAACCGATTCGACGCACATTGTCAAGCCCCTGC 400
P A K S Q S I L P D L T N F T F V R V E P C E T D S T H I V K P L L
401 TCCGAGGTGCTGACAAATGCTATCGCTATGAGAAGAAAGTGGATAACTTCGGACGCGTCTCCAAGTACACGTTCTGGGCCTCACAGGACGACGATAACAC 500
R G A D K C Y R Y E K K V D N F G R V S K Y T F W A S Q D D D N T
501 CCCAATTCCGGTACGCTACGTCATGATGGGTTACGACTCACTCTTGGGATCGCACTTCGACAAGTATGAGGTCGTGTACACCGACTACACACCCGGACCC 600
P I P V R Y V M M G Y D S L L G S H F D K Y E V V Y T D Y T P G P
601 GTCGAAGATGACCTCTTCCAAGTCAAGACTGTTATTGACAAGGAATGCACTTCGTTCCCGTCGCCGCCGGGCGTGTCCACTACCCACCTGTTCAACCCGA 700
V E D D L F Q V K T V I D K E C T S F P S P P G V S T T H L F N P M
701 TGGCGGAGTTCATTGACGAGAAGGATTCGCACGTGCACGAACACTTCGAGCACTTCAAATCGACACACGGCAAGGCATACGGCCACCAGGCCGAGGAGAT 800
A E F I D E K D S H V H E H F E H F K S T H G K A Y G H Q A E E I
801 CATCCGCAAGGACAATTTCCGCCACAACCAACGCTTCGTCAATTCGATGAACCGCCGCAACCTTTCGTACGCGCTGAAGCTCAACCACCGCGCCGACTGG 900
I R K D N F R H N Q R F V N S M N R R N L S Y A L K L N H R A D W
901 AGCCAGGACGAGTTCAGGCTGCTCCGGGGCCGTCTACAGTTCACCAGCCAGAAGTCGATGGCCAGGGAGTTCCCCAAGGAACAGTACTCGGATCGCGTCG 1000
S Q D E F R L L R G R L Q F T S Q K S M A R E F P K E Q Y S D R V E
1001 AGCCGGACTACGTCGACTGGCGACTCGAGGGAGCCGTCACGCCGGTCAAGGACCAGGCTGTGTGCGGGTCGTGCTGGAGCTTTGGCACGGTCGGCCACAT 1100
P D Y V D W R L E G A V T P V K D Q A V C G S C W S F G T V G H I
1101 CGAAGGCGCCTACTTCCGCAAGTTCGGCGAGCTGGTCCGTTTCTCCGAGCAGCAGCTCGTAGACTGTTCGTGGAATGCCGGCAACGATGCCTGCGACGGT 1200
E G A Y F R K F G E L V R F S E Q Q L V D C S W N A G N D A C D G
1201 GGTCTGGACTTTATCGCCTACCACTACATCCAGAAGTACGGACTGGCCAGCAACGACCAATACGGACCCTACCGCGGCATTGACGGCAAATGCAAGGACC 1300
G L D F I A Y H Y I Q K Y G L A S N D Q Y G P Y R G I D G K C K D L
1301 TGGAGATTTCCAACAAGCCCATTAGCACGCTGAAAGGCTACCGAAACGTGACCACTGTGGAAGACCTCCGCAAGGCGCTCGCGTTTGTCGGCCCCATATC 1400
E I S N K P I S T L K G Y R N V T T V E D L R K A L A F V G P I S
1401 GGTGTCGATCGATGCATCAAGGCCGTCGCTCAGCTTCTATTCGCATGGAGTCTACAGCGATCCGGACTGCAGTTCGACGGAACTCGACCACTCCGTGCTC 1500
V S I D A S R P S L S F Y S H G V Y S D P D C S S T E L D H S V L
1501 GCTGTTGGCTGCGGCACGCTGCACGGTGAGCCGTACTGGCTCATCAAGAACTCGTGGTCCACGTACTGGGGCAACGACGGATACATTCTCATCTCGCAGA 1600
A V G Y G T L H G E P Y W L I K N S W S T Y W G N D G Y I L I S Q K
1601 AGGACAACATGTGCGGCGTTGCCTCGCAGGCAACCTACGTCGAGCTGTAG 1650
D N M C G V A S Q A T Y V E L *
(B)
PRM cysteine protease
Signal peptide
Inhibitor
Peptidase
N
C
1
18
249
305
335
547
549
Peptidase domain fused with histidine-tag
6xHis
Inhibitor
Peptidase
N
C
Peptidase domain fused with histidine-tagged trigger factor protein
6xHis
Trigger Factor
Peptidase
N
C
Whole region without signal peptide fused with histidine-tagged trigger factor protein
6xHis
Trigger Factor
Inhibitor
Peptidase
N
C
