# Supplementary figures and images for: Characterisation of a cysteine protease from poultry red mites and its potential use as a vaccine for chickens
Source: Parasite. 2021 Feb 3;28:9. doi: 10.1051/parasite/2021005 (PMC7863971; doi:10.1051/parasite/2021005)

(kD)

M

Insoluble fraction

Soluble fraction

M

Soluble fraction

Purified protein

150

100

75

50

37

25

20

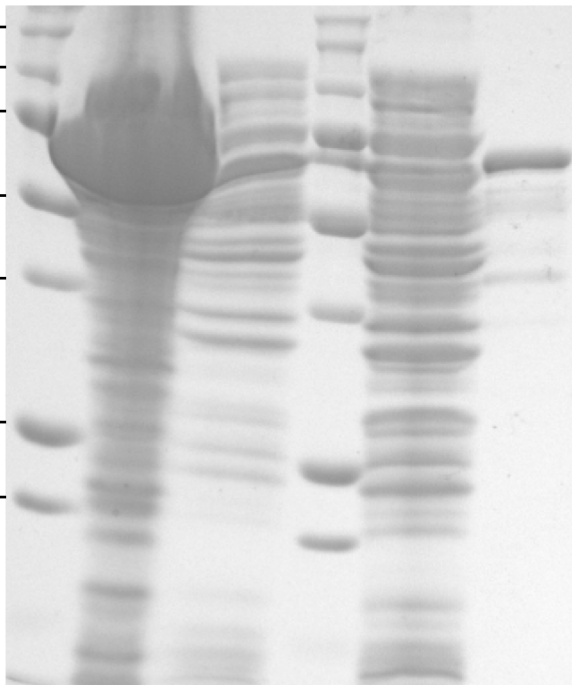

◀ Deg-CPR-1

Supplement: Supplementary file 3 — Supplementary Figure 2. The expression and purification of cysteine protease from poultry red mites (PRMs, Dermanyssus gallinae) (Deg-CPR-1). The entire recombinant Deg-CPR-1 without the signal peptides, fused with the histidine tag, was expressed and purified for immunisation. The recombinant Deg-CPR-1 was purified from the insoluble inclusion body. The recombinant Deg-CPR-1 was extracted from the insoluble inclusion body and purified using the metal affinity resins. M: Marker (Precision Plus Protein™ All Blue Prestained Protein Standards, Bio-Rad, Hercules, CA, USA). [file parasite-28-9-s2.pdf]

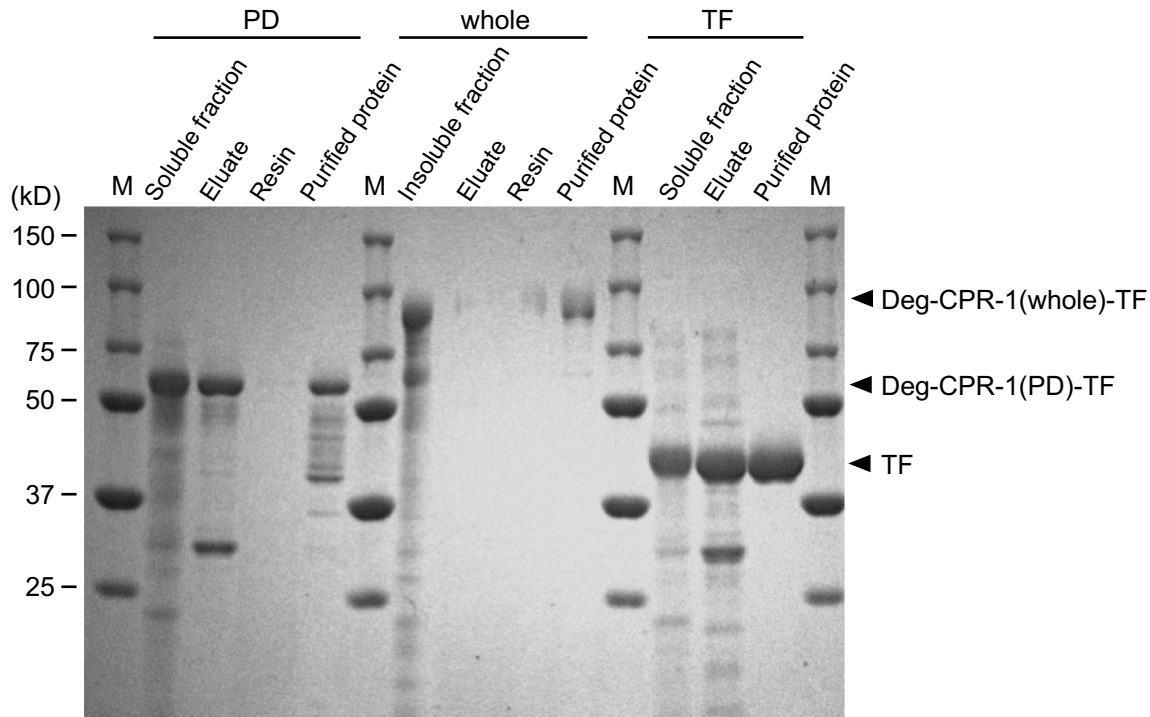

Supplement: Supplementary file 4 — Supplementary Figure 3. The expression and purification of cysteine protease from poultry red mites (PRMs, Dermanyssus gallinae) (Deg-CPR-1) fused with histidine-tagged trigger factor (TF). Two recombinant Deg-CPR-1 proteins fused with histidine-tagged TF were used. The peptidase domain fused with histidine-tagged TF (Deg-CPR-1(PD)-TF), the entire region without signal peptides fused with histidine-tagged TF (Deg-CPR-1(whole)-TF), and TF were expressed and purified for the analysis of enzyme activity. Deg-CPR-1(PD)-TF and TF were purified from the soluble fraction, and Deg-CPR-1(whole)-TF was purified from the insoluble inclusion body. The recombinant proteins were purified using the metal affinity resins. After the elution of recombinant proteins from the resins, the buffer was changed to phosphate-buffered saline by ultrafiltration. M: Marker (Precision Plus Protein™ All Blue Prestained Protein Standards, Bio-Rad). [file parasite-28-9-s3.pdf]
